# Supplementary material for: Weight Pulling: A Novel Mouse Model of Human Progressive Resistance Exercise
Source: Cells. 2021 Sep 17;10(9):2459. doi: 10.3390/cells10092459 (PMC8465477; doi:10.3390/cells10092459)
Supplement: Supplementary file 1 [file cells-10-02459-s001.zip › cells-1393998 (4).pdf]

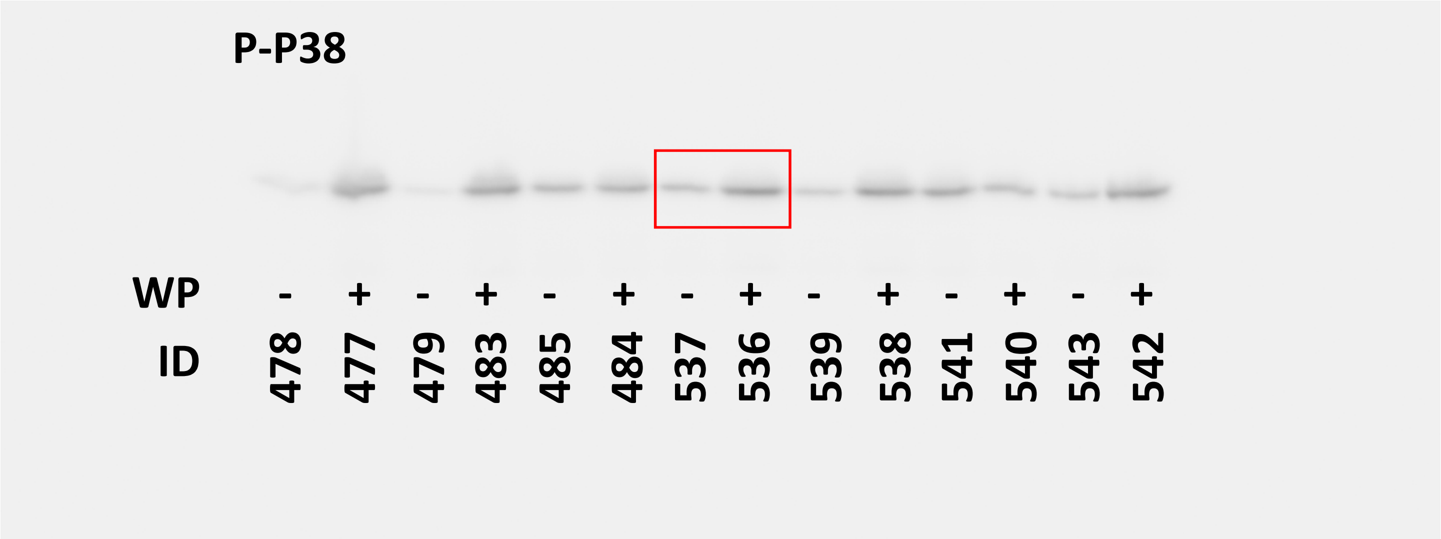
P-P38
WP - + - + - + - + - + - + - +
ID 478 477 479 483 485 484 537 536 539 538 541 540 543 542

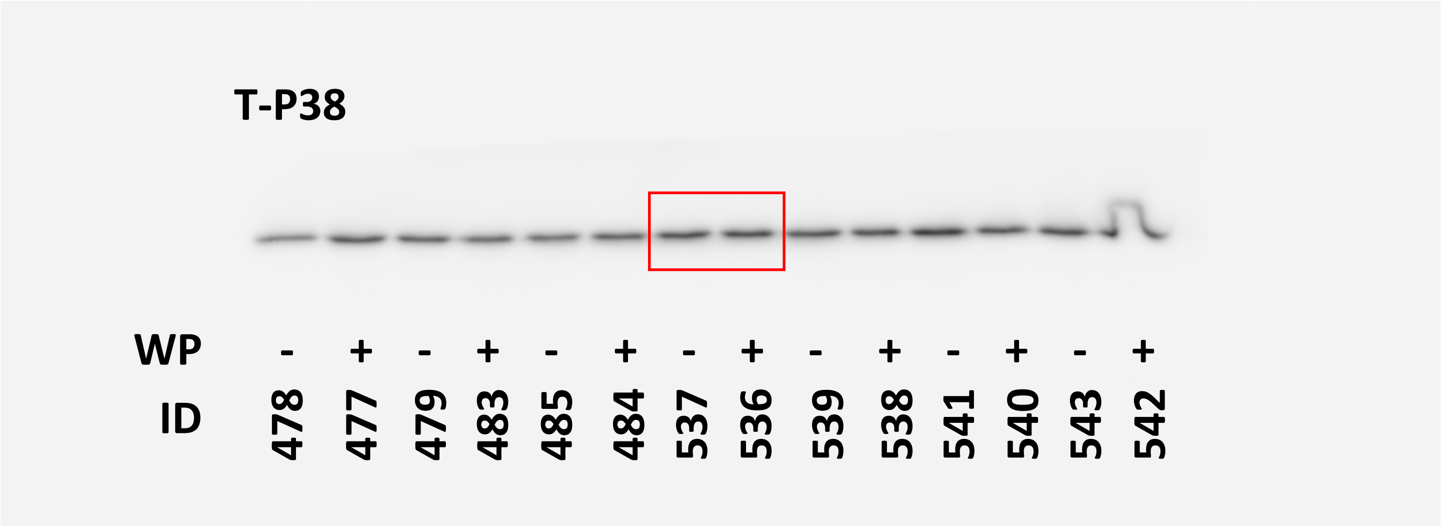
T-P38
WP - + - + - + - + - + - + - +
ID 478 477 479 483 485 484 537 536 539 538 541 540 543 542

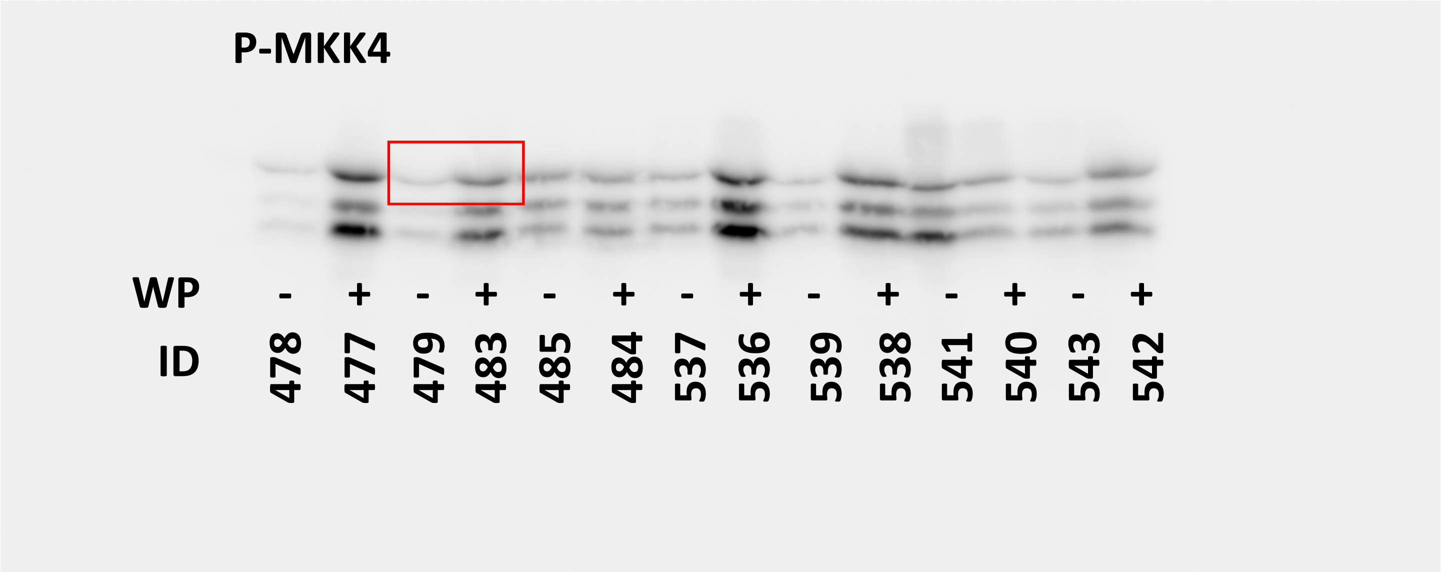
P-MKK4
WP - + - + - + - + - + - + - +
ID 478 477 479 483 485 484 537 536 539 538 541 540 543 542

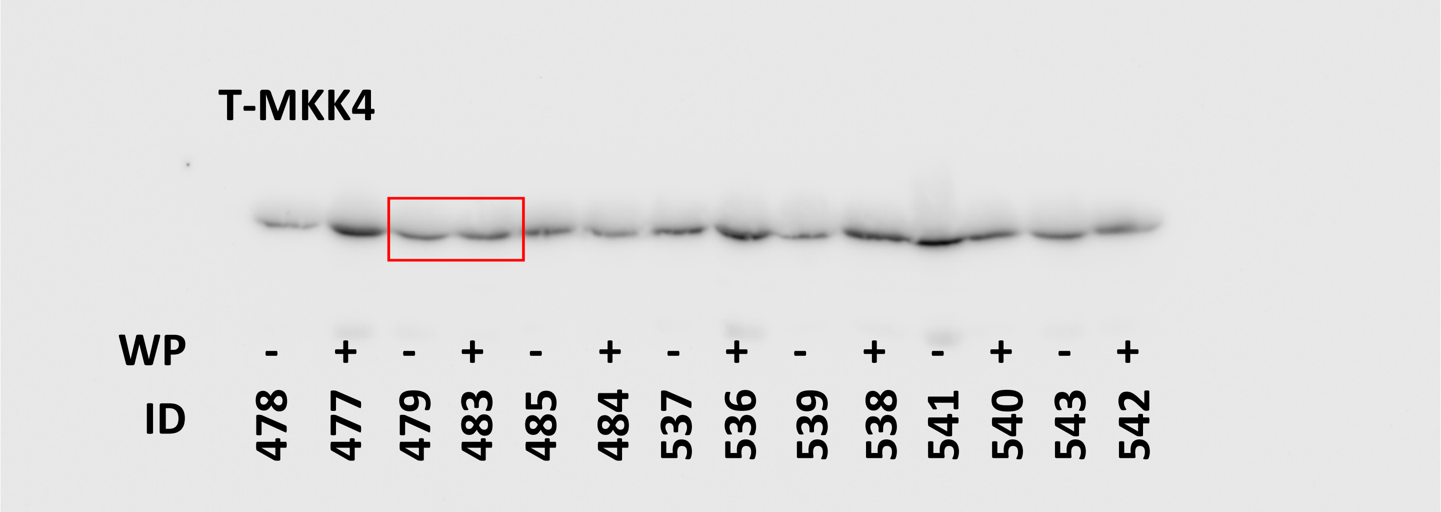
T-MKK4
WP - + - + - + - + - + - + - +
ID 478 477 479 483 485 484 537 536 539 538 541 540 543 542

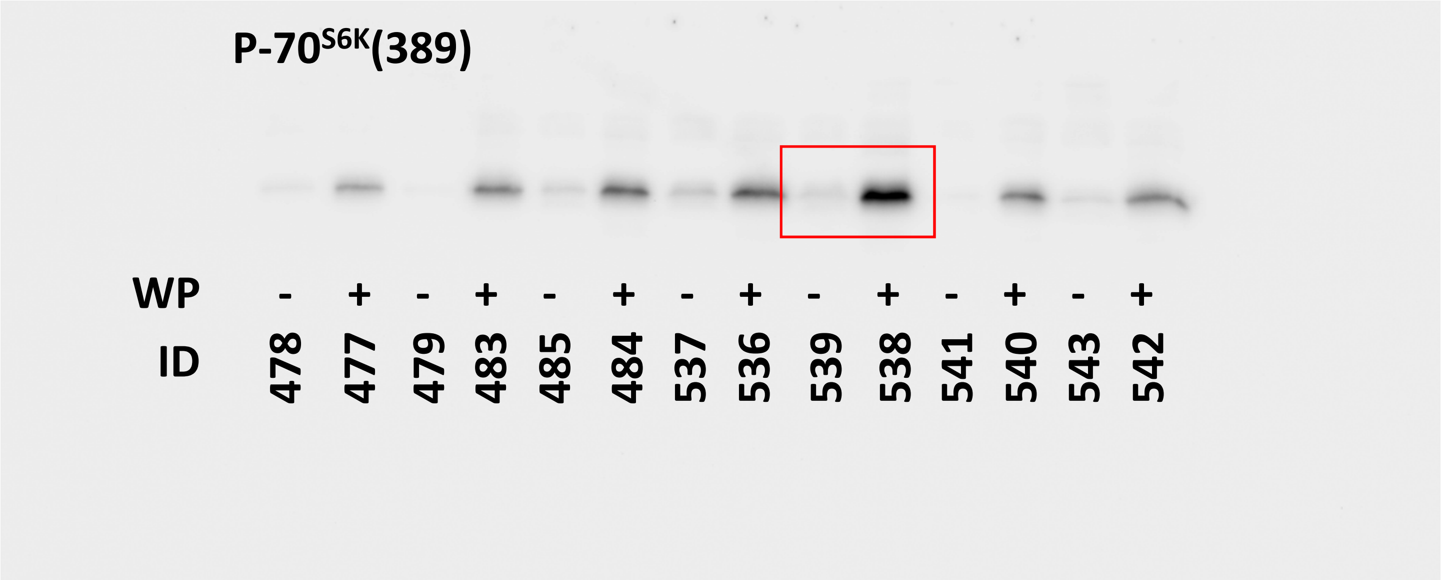
P-70S6K(389)
WP - + - + - + - + - + - + - +
ID 478 477 479 483 485 484 537 536 539 538 541 540 543 542

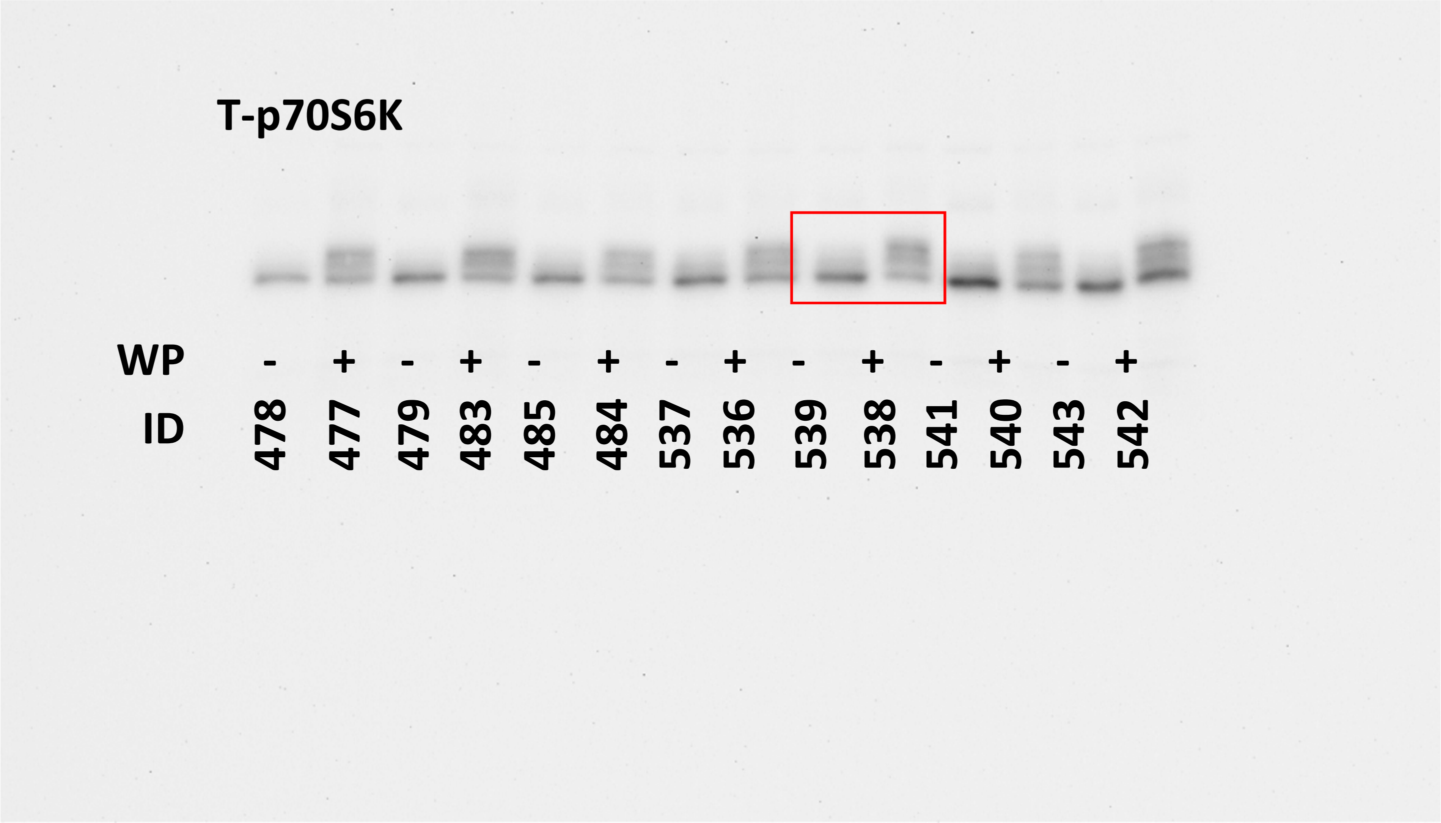
T-p70S6K
WP - + - + - + - + - + - + - +
ID 478 477 479 483 485 484 537 536 539 538 541 540 543 542

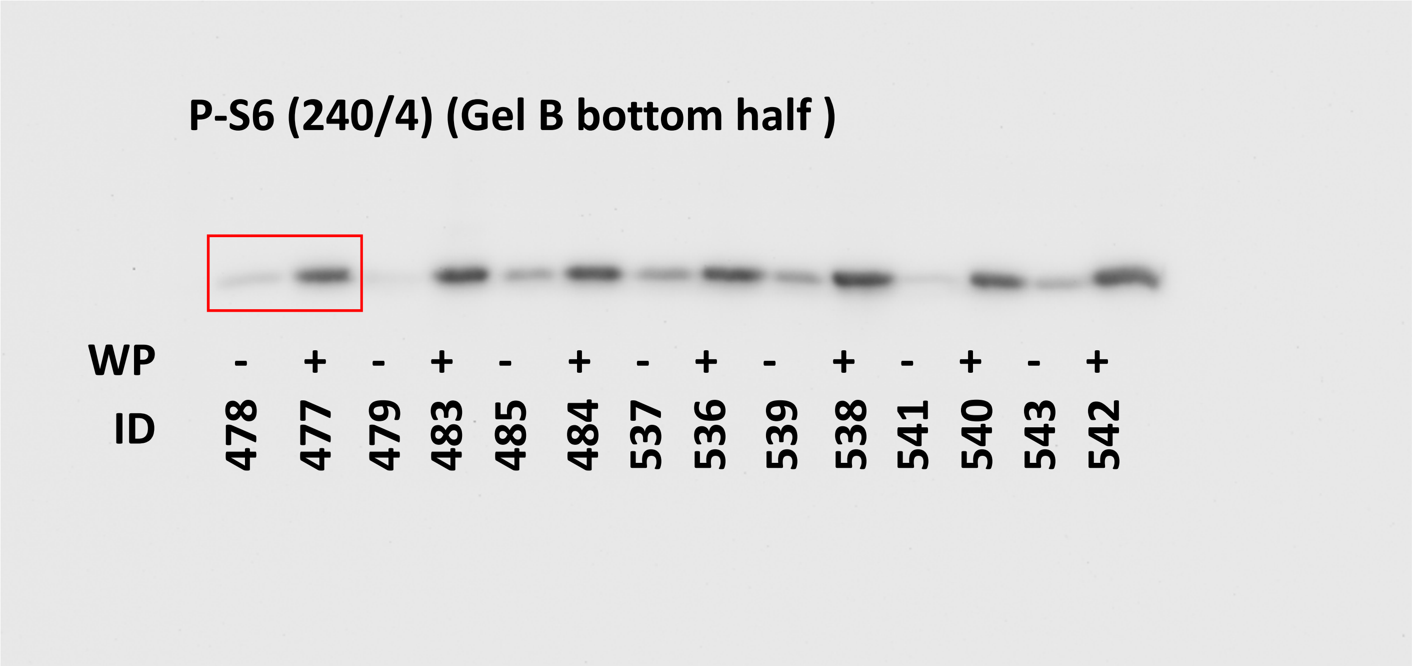
P-S6 (240/4) (Gel B bottom half)
WP - + - + - + - + - + - + - +
ID 478 477 479 483 485 484 537 536 539 538 541 540 543 542

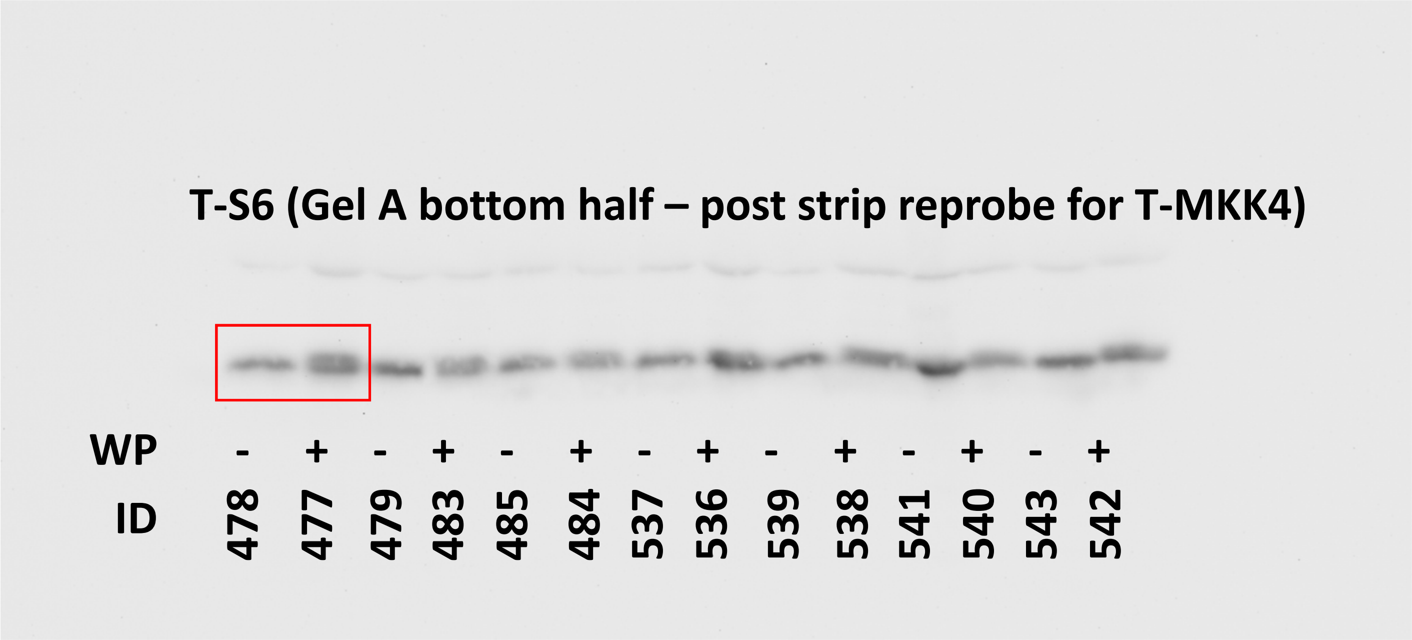
T-S6 (Gel A bottom half – post strip reprobe for T-MKK4)
WP - + - + - + - + - + - + - +
ID 478 477 479 483 485 484 537 536 539 538 541 540 543 542

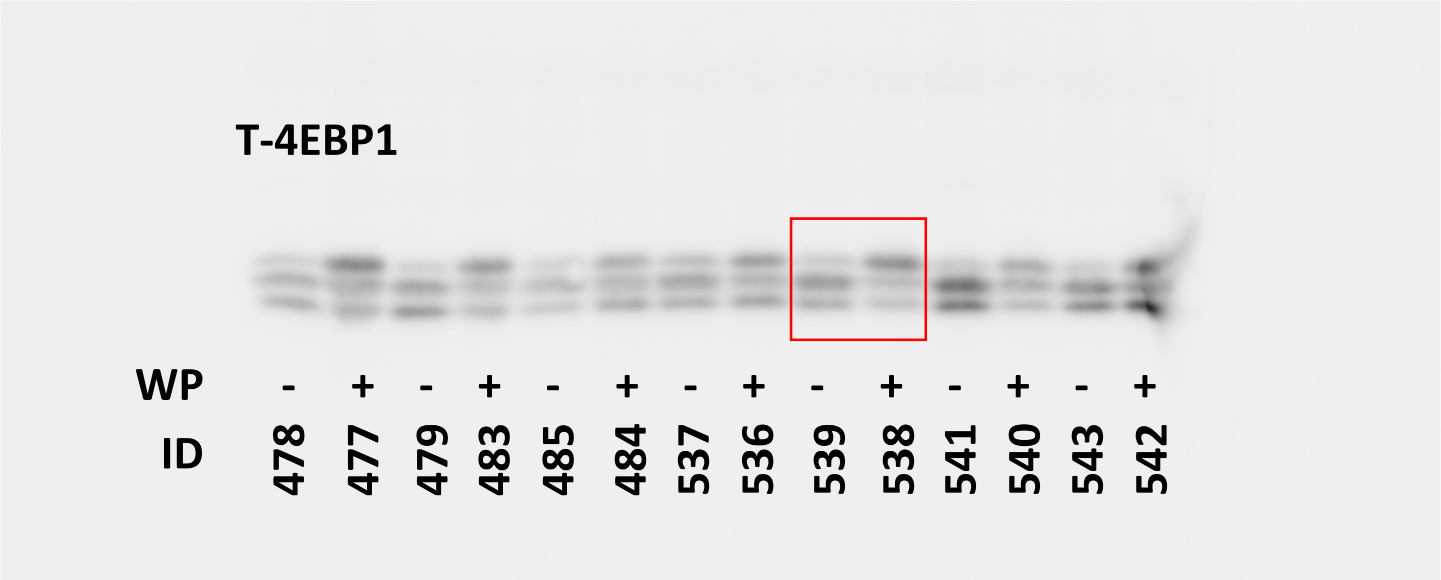
T-4EBP1
WP - + - + - + - + - + - + - +
ID 478 477 479 483 485 484 537 536 539 538 541 540 543 542

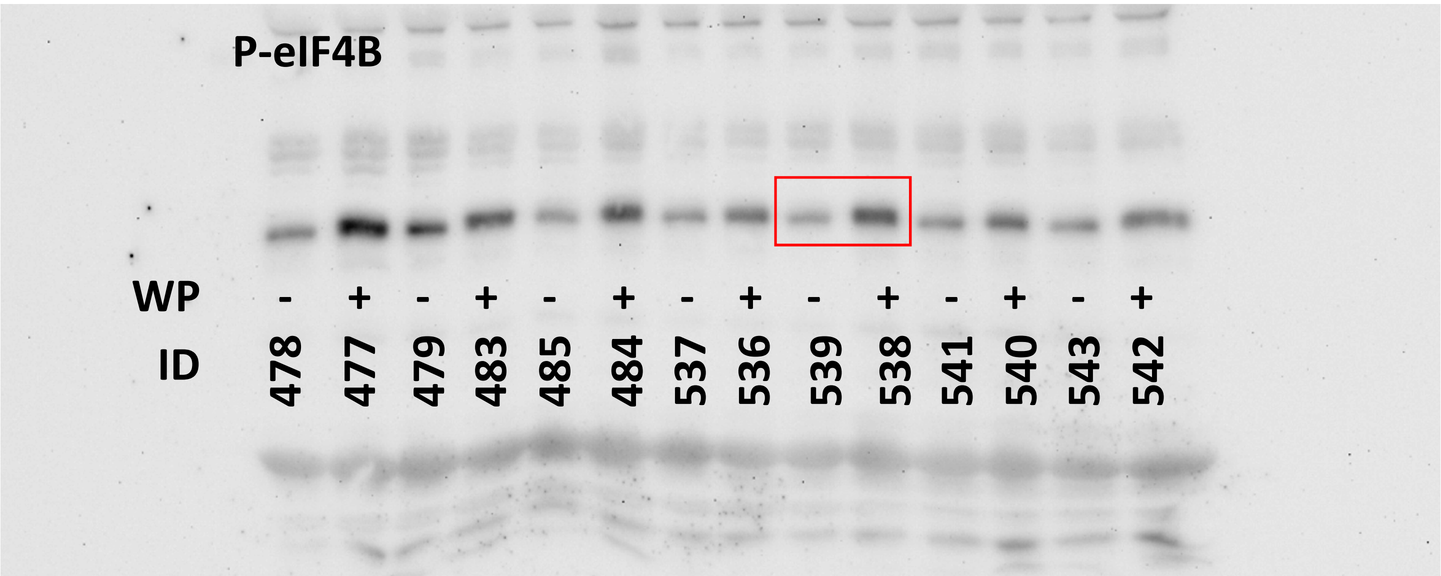
P-eIF4B
WP - + - + - + - + - + - + - +
ID 478 477 479 483 485 484 537 536 539 538 541 540 543 542

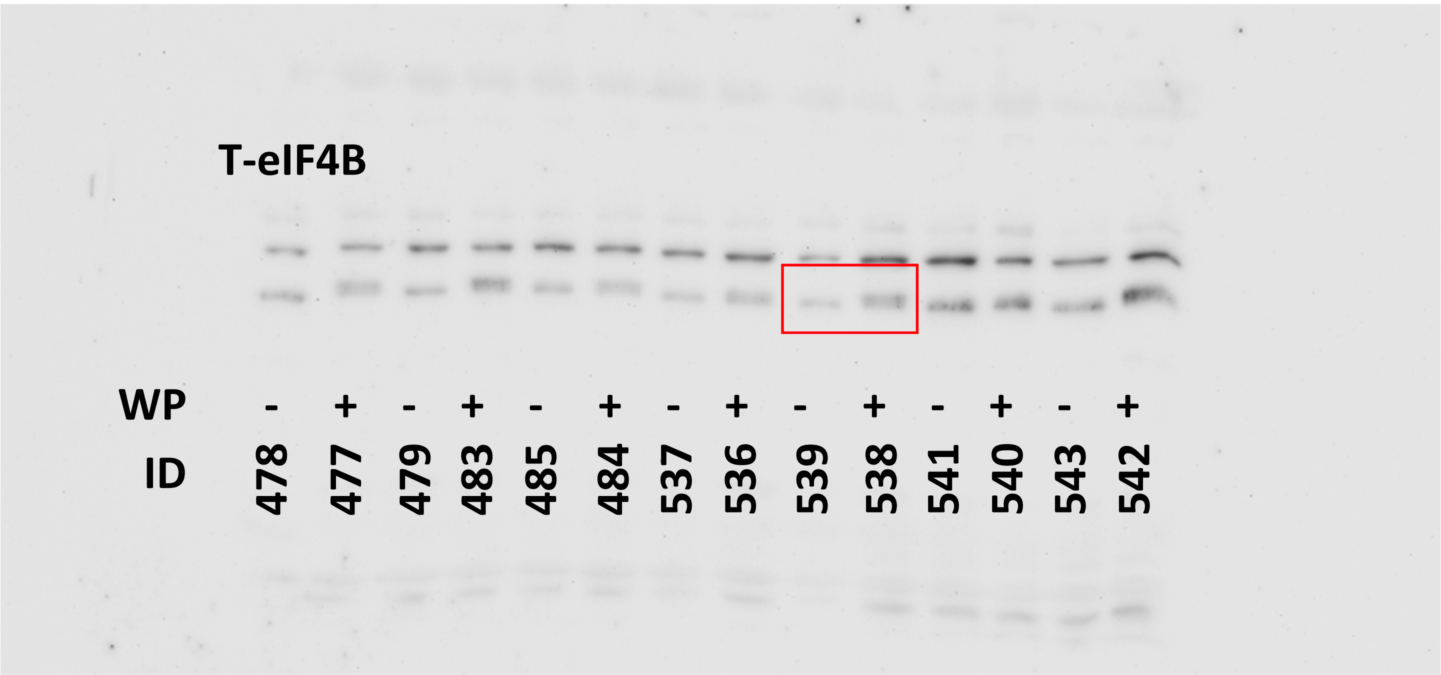
T-eIF4B
WP - + - + - + - + - + - + - +
ID 478 477 479 483 485 484 537 536 539 538 541 540 543 542

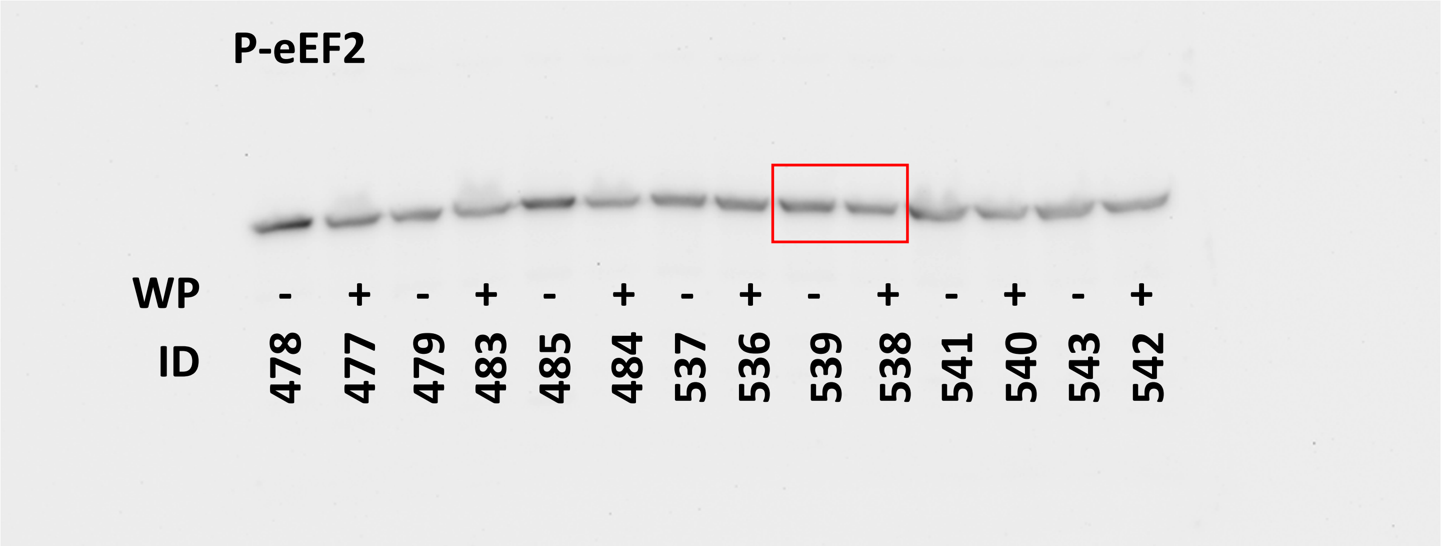
P-eEF2
WP - + - + - + - + - + - + - +
ID 478 477 479 483 485 484 537 536 539 538 541 540 543 542

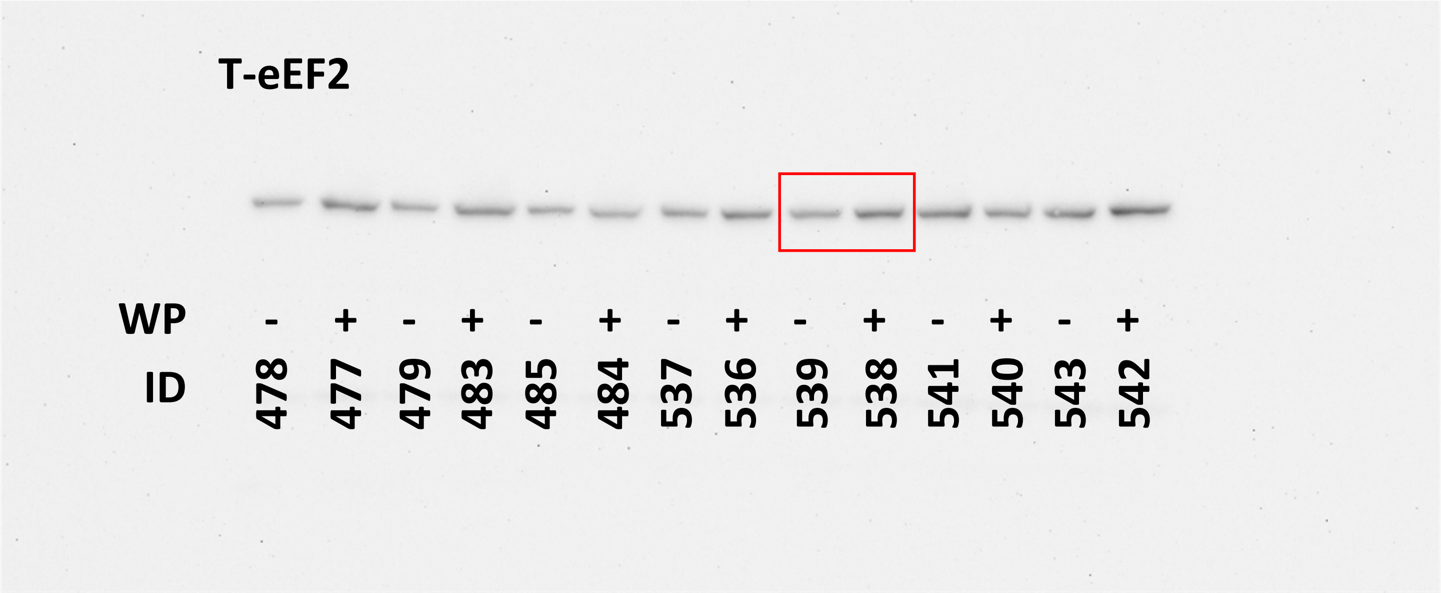
T-eEF2
WP - + - + - + - + - + - + - +
ID 478 477 479 483 485 484 537 536 539 538 541 540 543 542

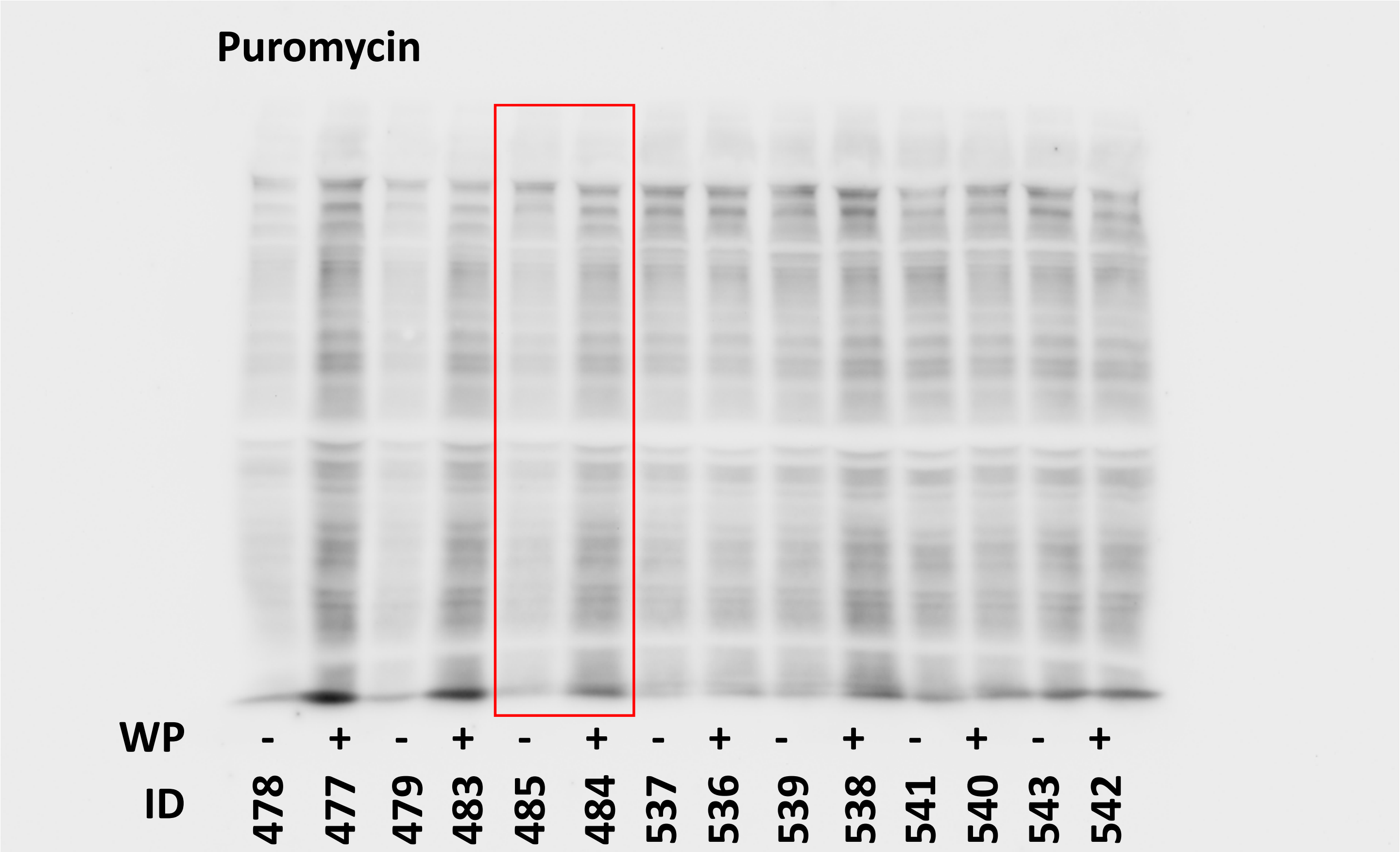
Puromycin
WP - + - + - + - + - + - + - +
ID 478 477 479 483 485 484 537 536 539 538 541 540 543 542

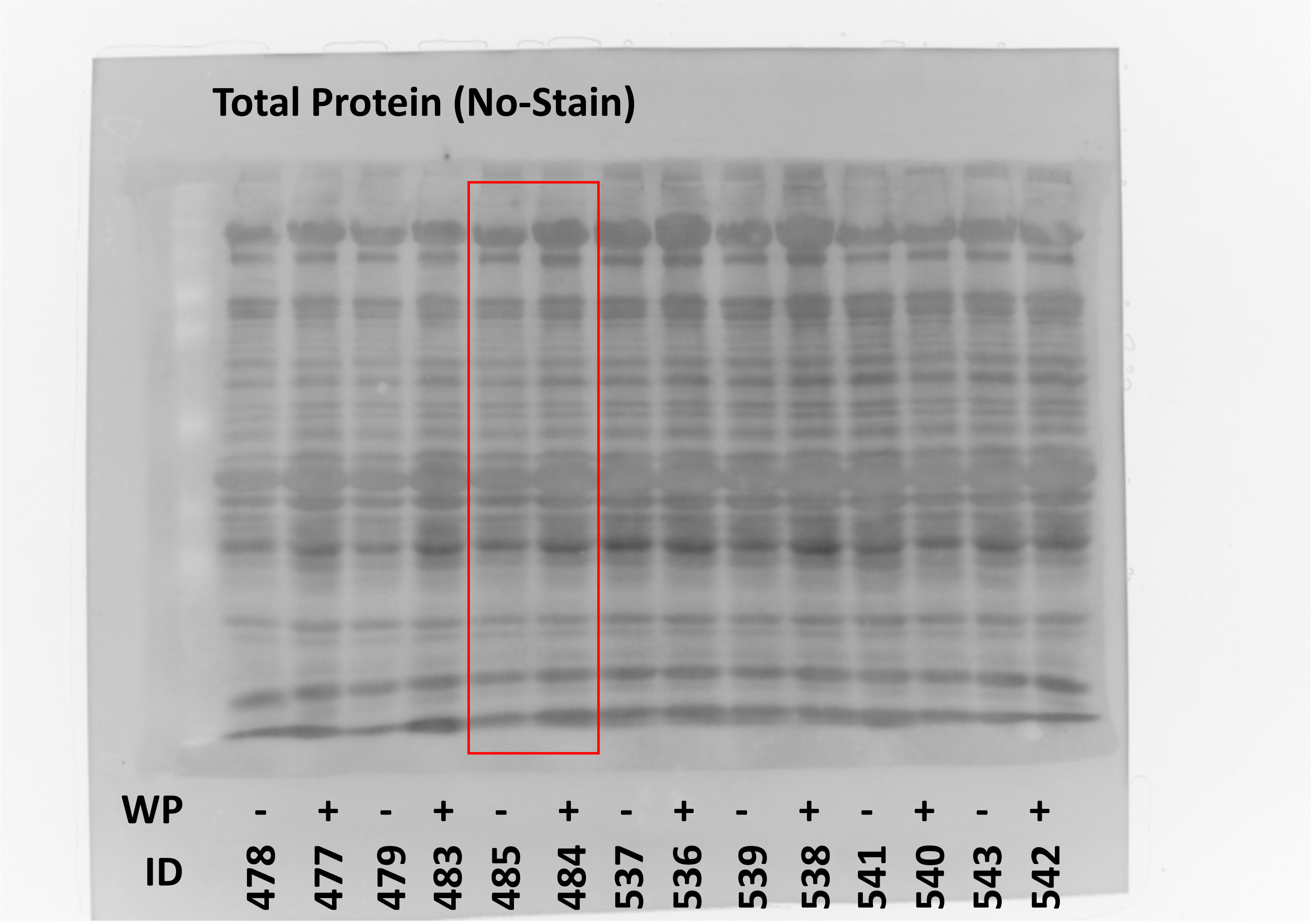
Total Protein (No-Stain)
WP - + - + - + - + - + - + - +
ID 478 477 479 483 485 484 537 536 539 538 541 540 543 542
