# Supplementary figures and images for: Weight Pulling: A Novel Mouse Model of Human Progressive Resistance Exercise
Source: Cells. 2021 Sep 17;10(9):2459. doi: 10.3390/cells10092459 (PMC8465477; doi:10.3390/cells10092459)

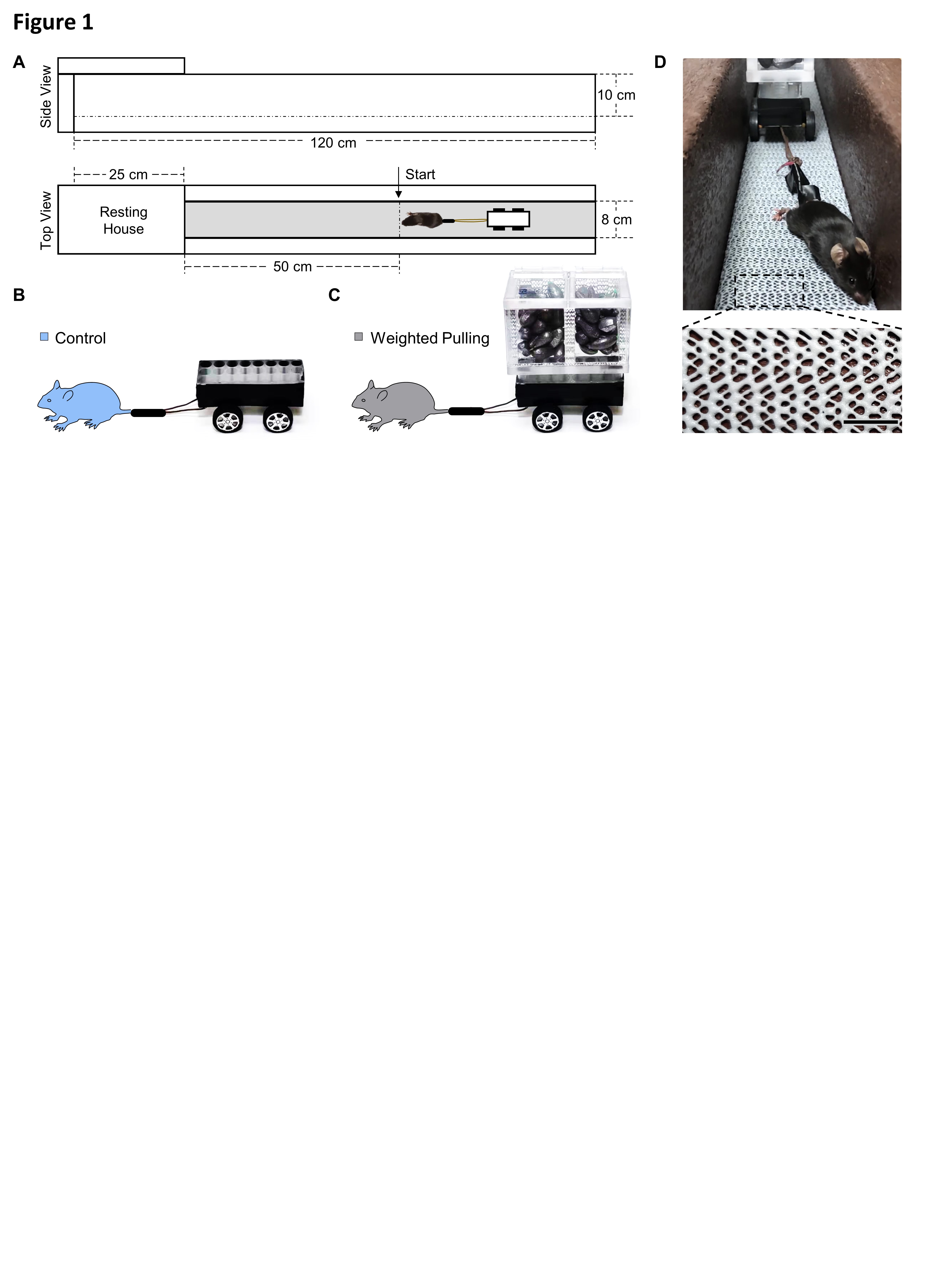

Supplement: Supplementary file 1 [file cells-10-02459-s001.zip › Figure 1.jpg]

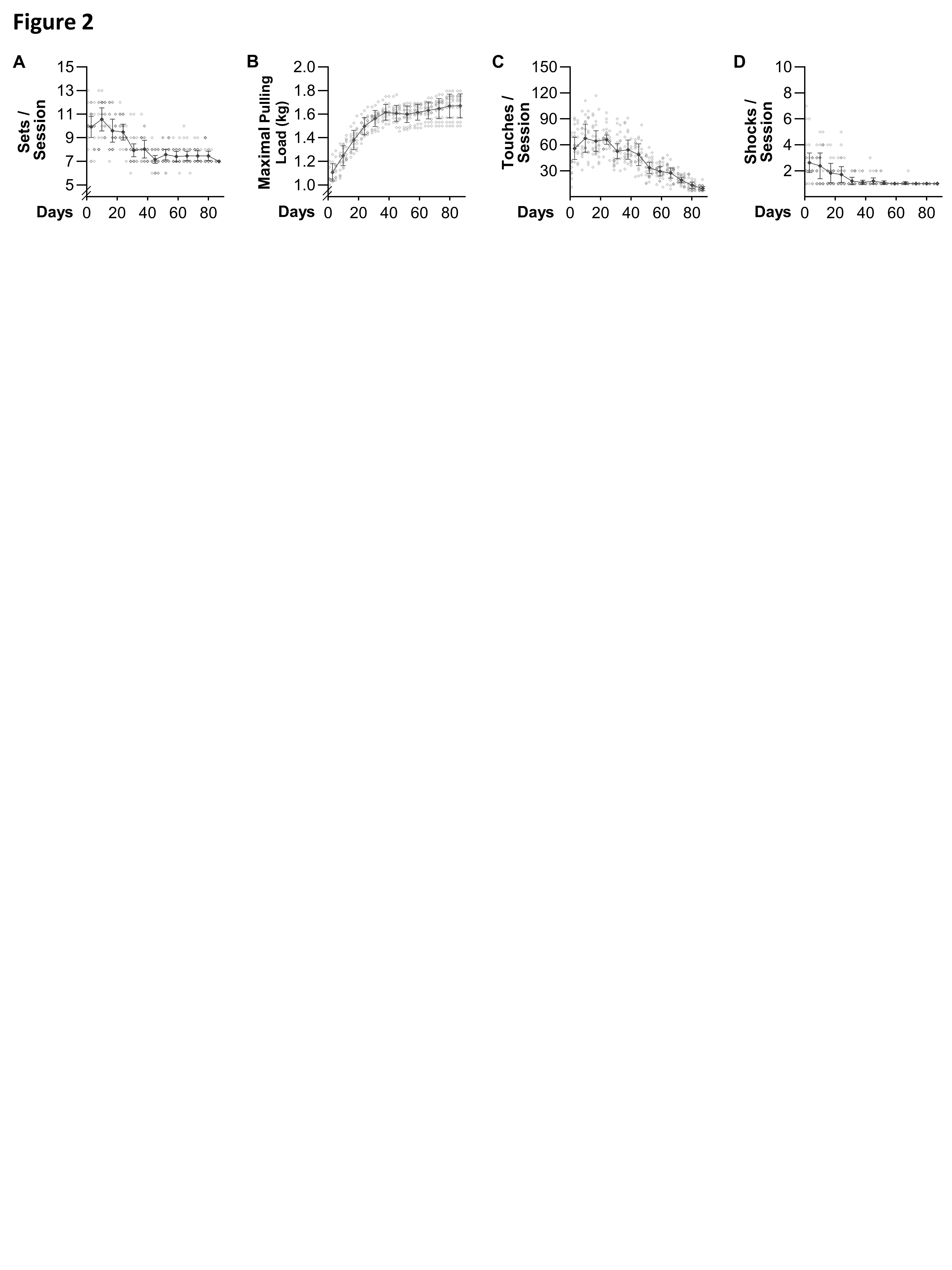

Supplement: Supplementary file 1 [file cells-10-02459-s001.zip › Figure 2.jpg]

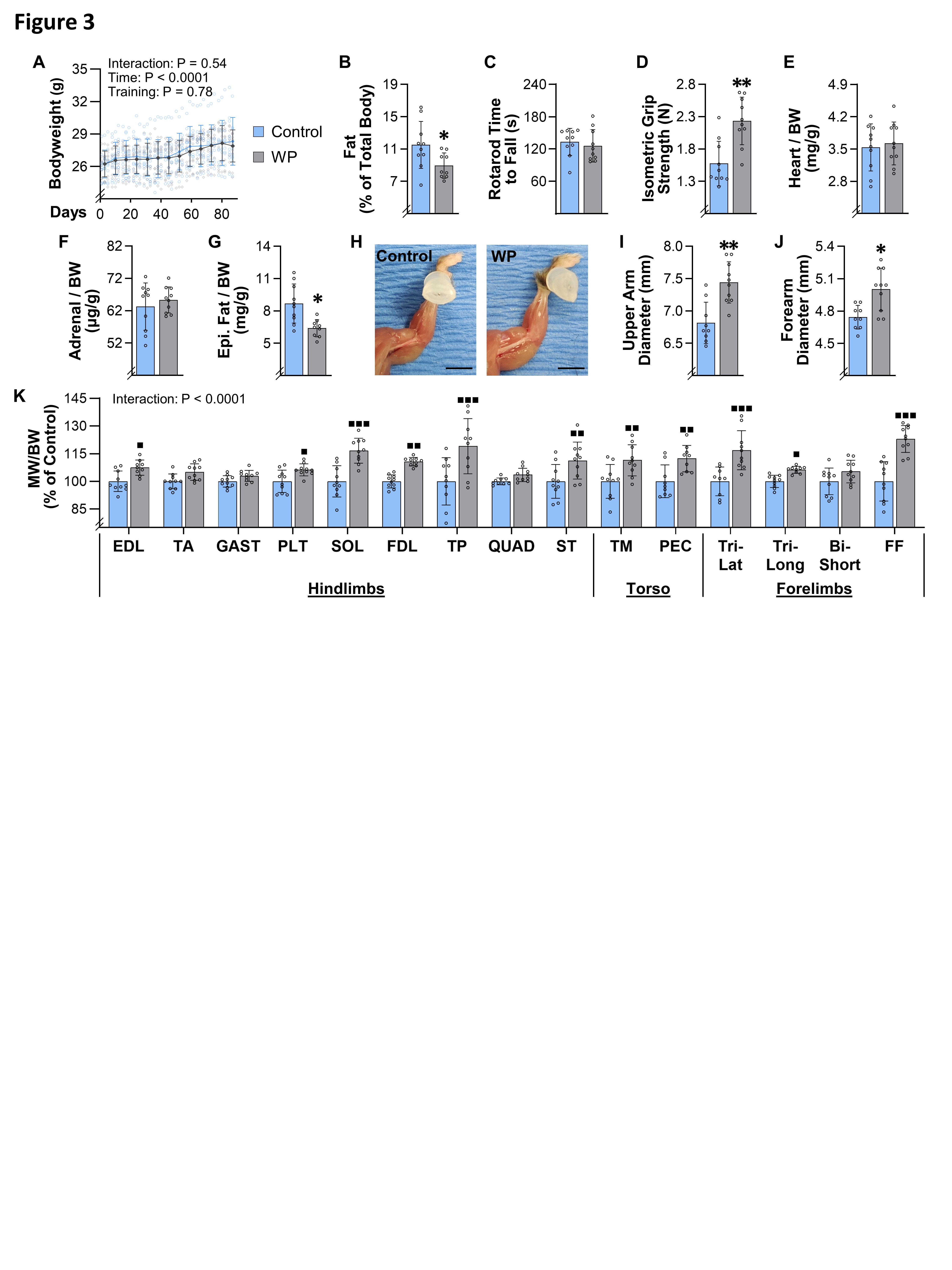

Supplement: Supplementary file 1 [file cells-10-02459-s001.zip › Figure 3.jpg]

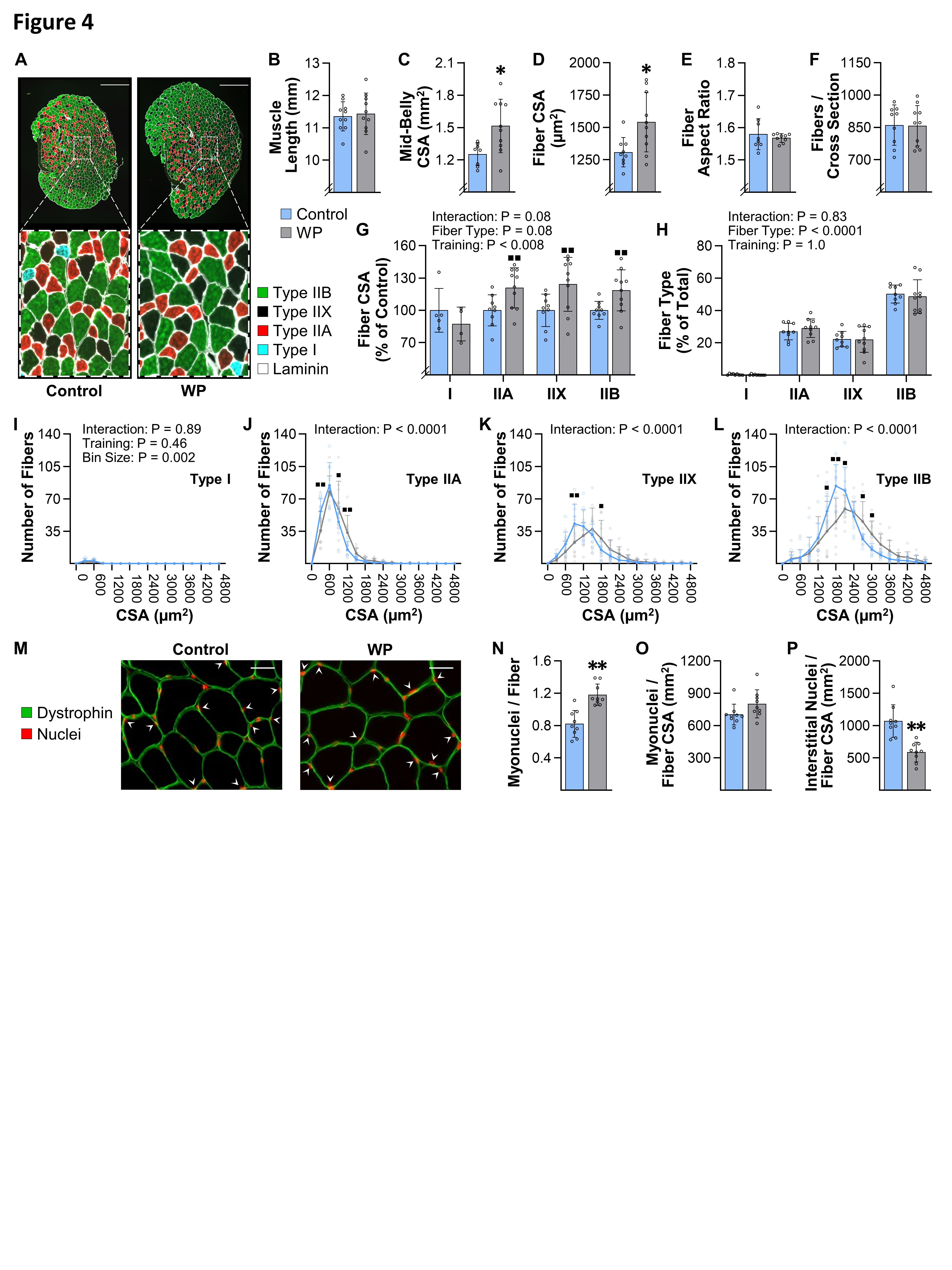

Supplement: Supplementary file 1 [file cells-10-02459-s001.zip › Figure 4.jpg]

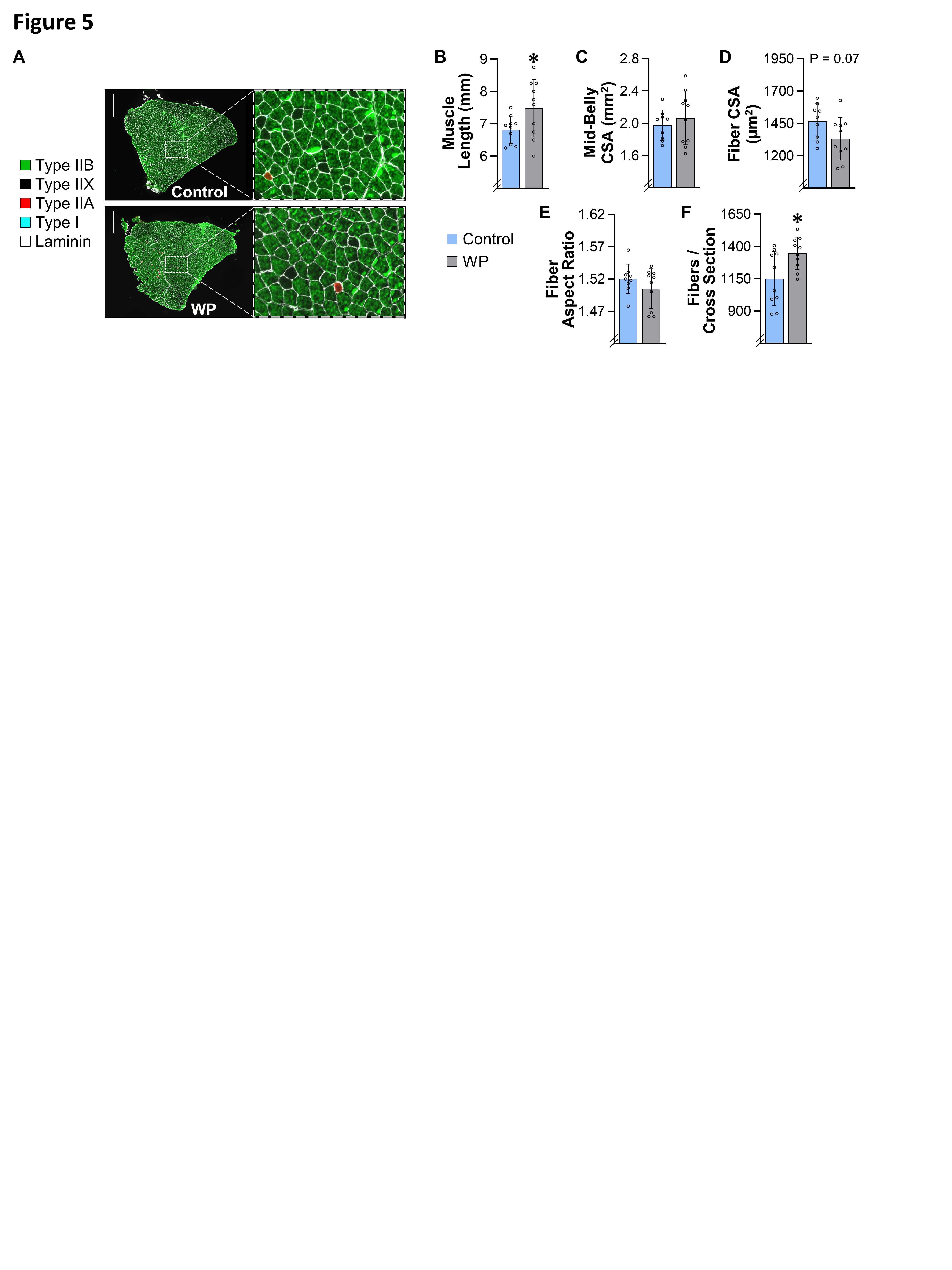

Supplement: Supplementary file 1 [file cells-10-02459-s001.zip › Figure 5.jpg]

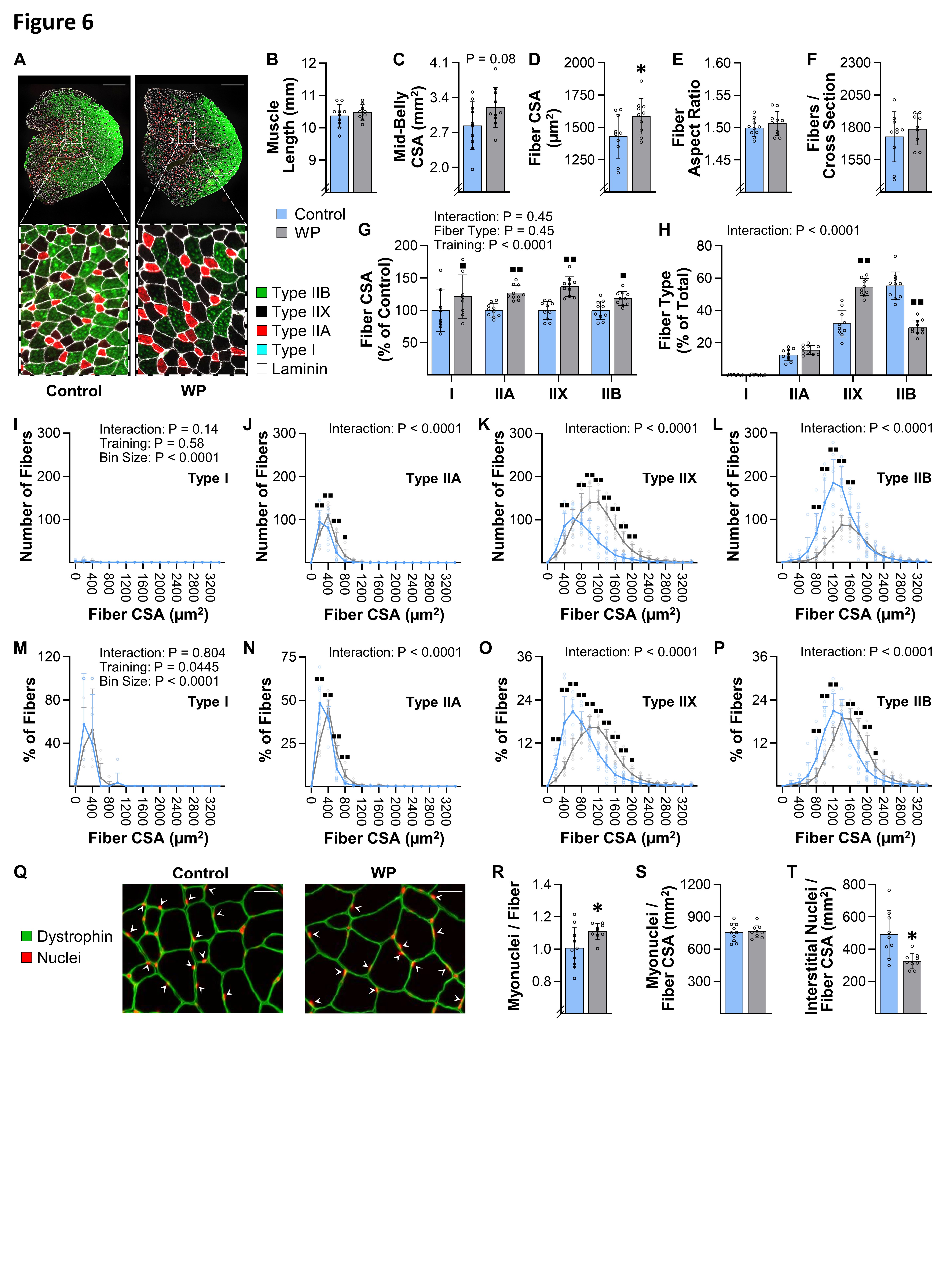

Supplement: Supplementary file 1 [file cells-10-02459-s001.zip › Figure 6.jpg]

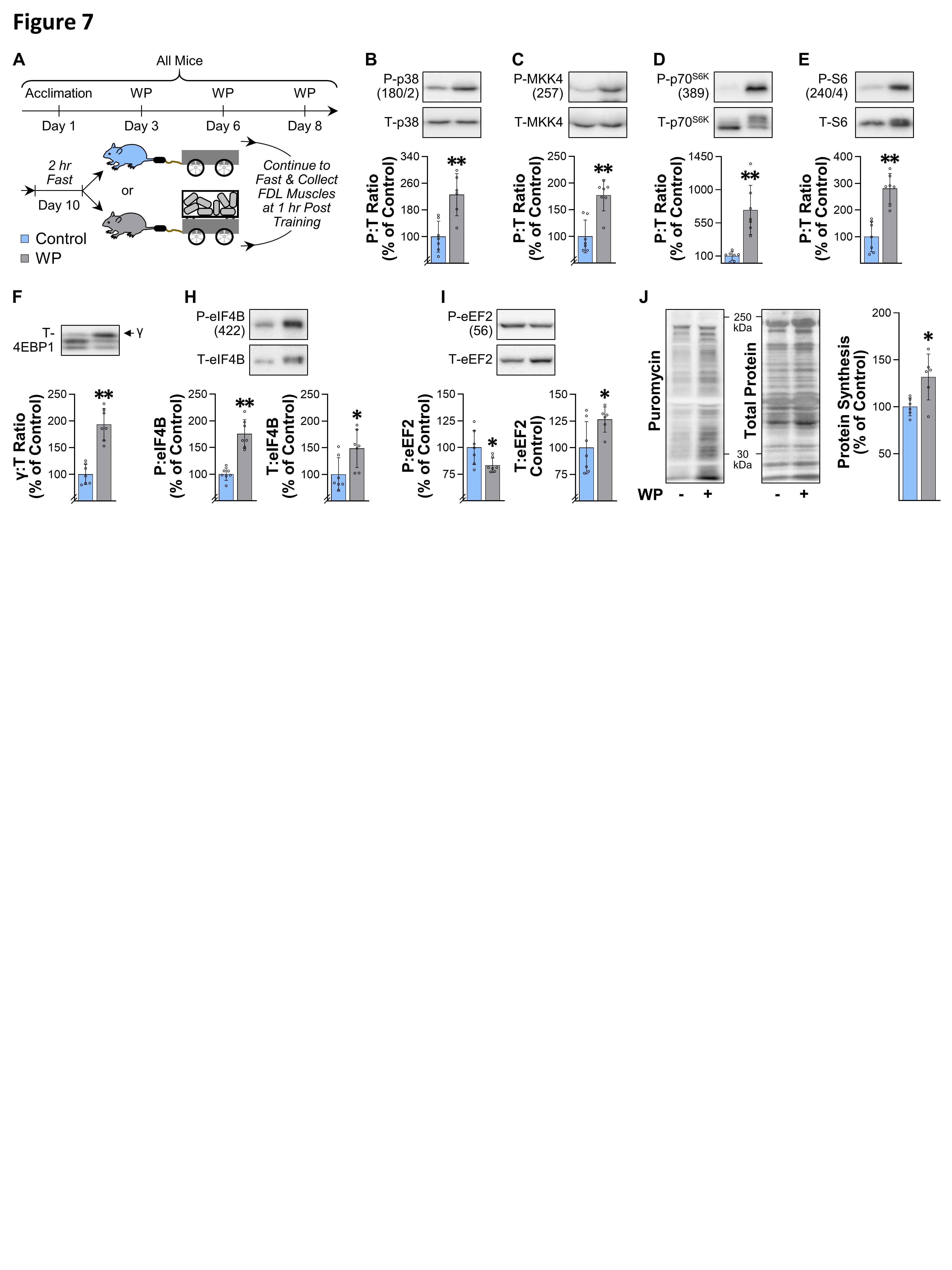

Supplement: Supplementary file 1 [file cells-10-02459-s001.zip › Figure 7.jpg]

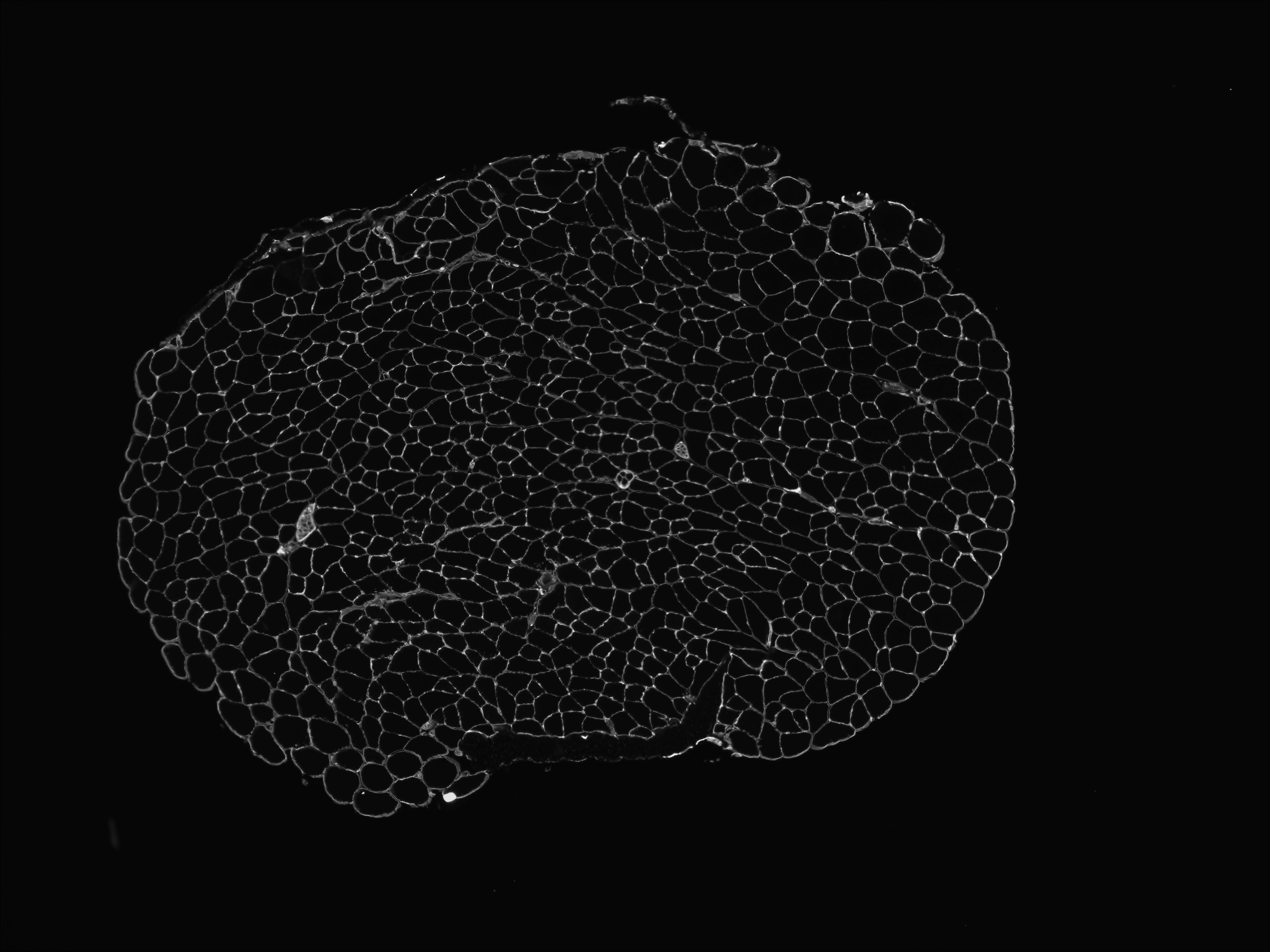

Supplement: Supplementary file 1 [file cells-10-02459-s001.zip › Supplemental Methods/Fiber Cross-Sectional Area Pipeline/Sample Images for Fiber CSA Measurements/Ch0.tif]

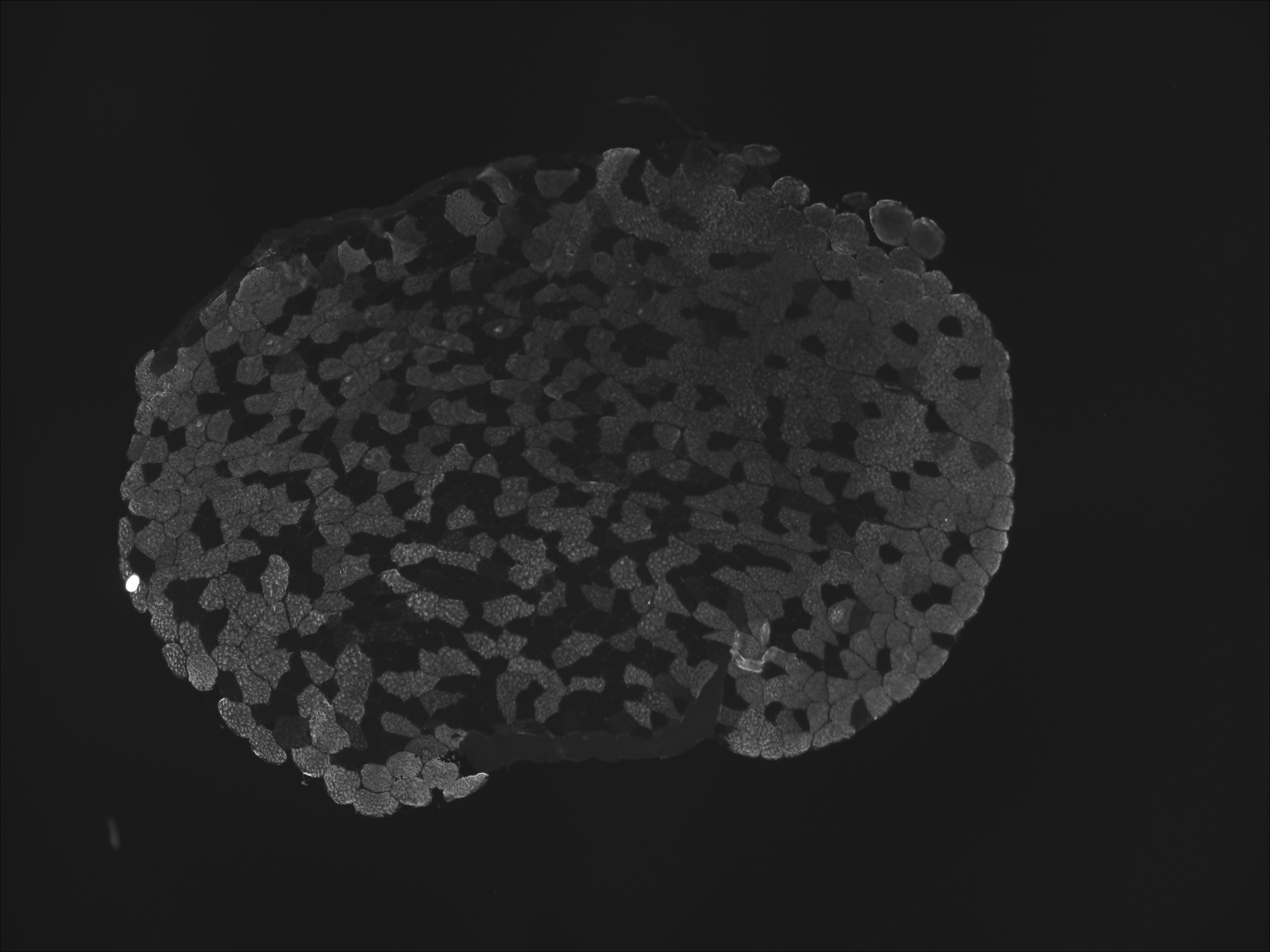

Supplement: Supplementary file 1 [file cells-10-02459-s001.zip › Supplemental Methods/Fiber Cross-Sectional Area Pipeline/Sample Images for Fiber CSA Measurements/Ch1.tif]

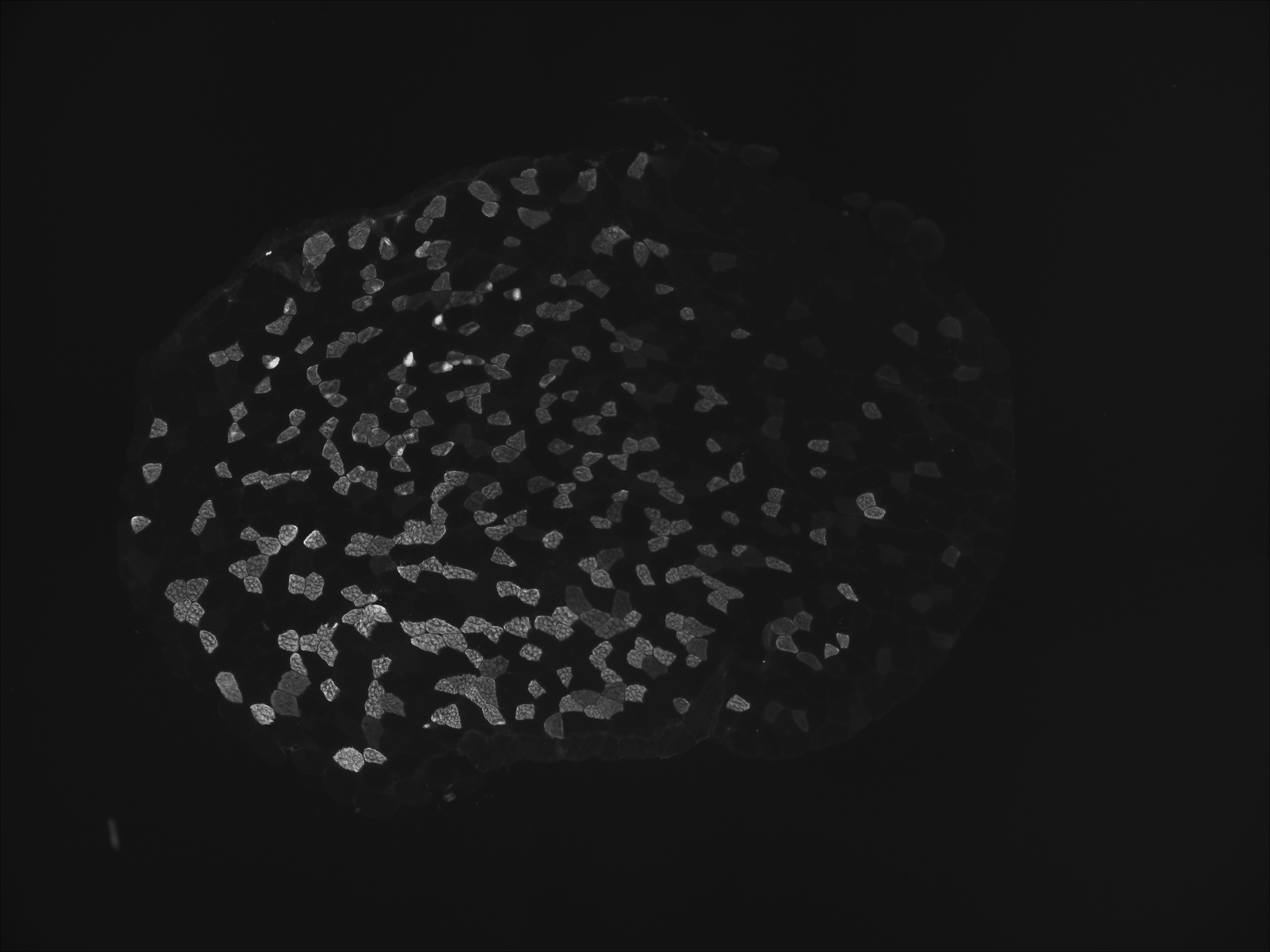

Supplement: Supplementary file 1 [file cells-10-02459-s001.zip › Supplemental Methods/Fiber Cross-Sectional Area Pipeline/Sample Images for Fiber CSA Measurements/Ch2.tif]

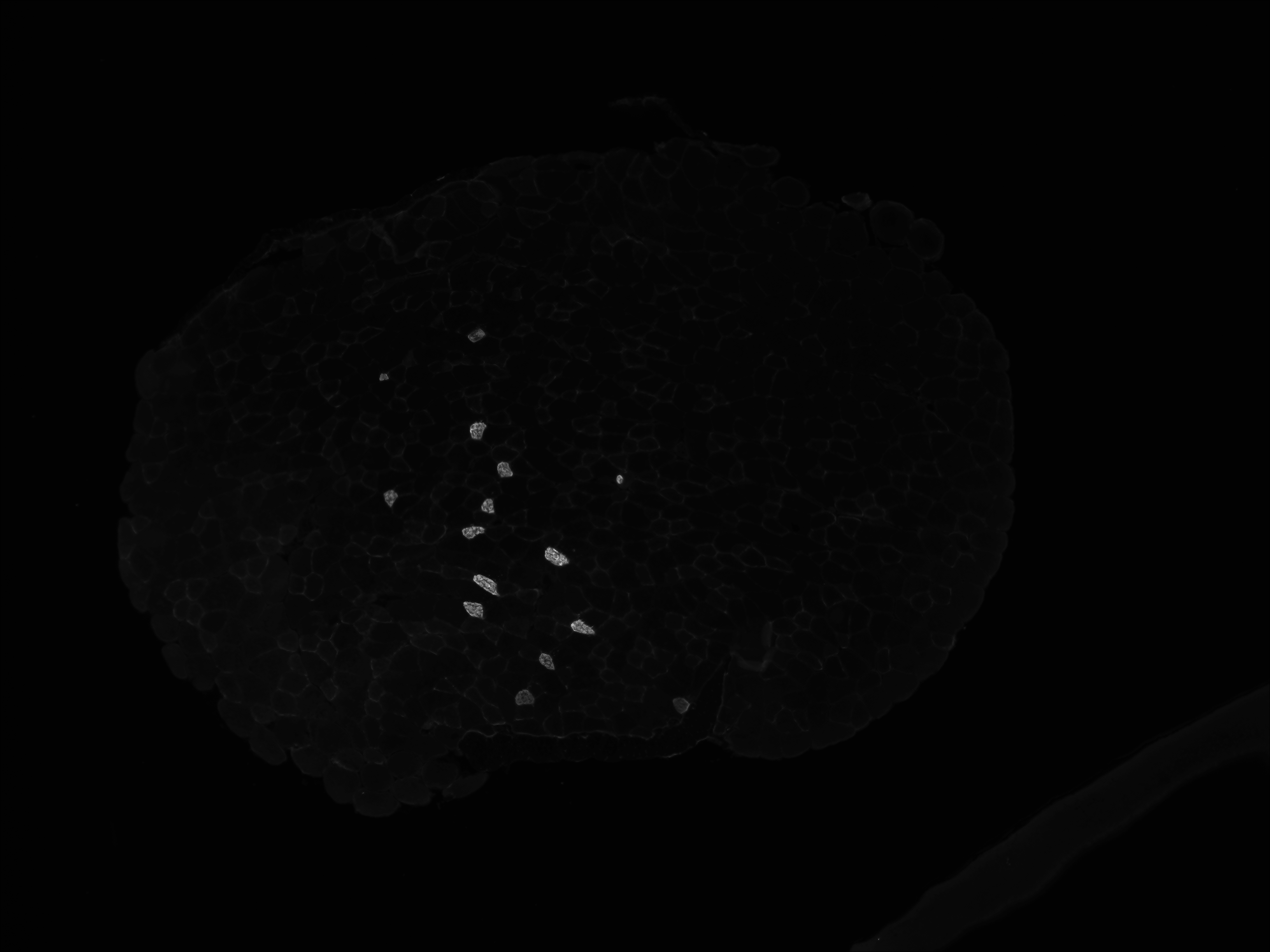

Supplement: Supplementary file 1 [file cells-10-02459-s001.zip › Supplemental Methods/Fiber Cross-Sectional Area Pipeline/Sample Images for Fiber CSA Measurements/Ch3.tif]

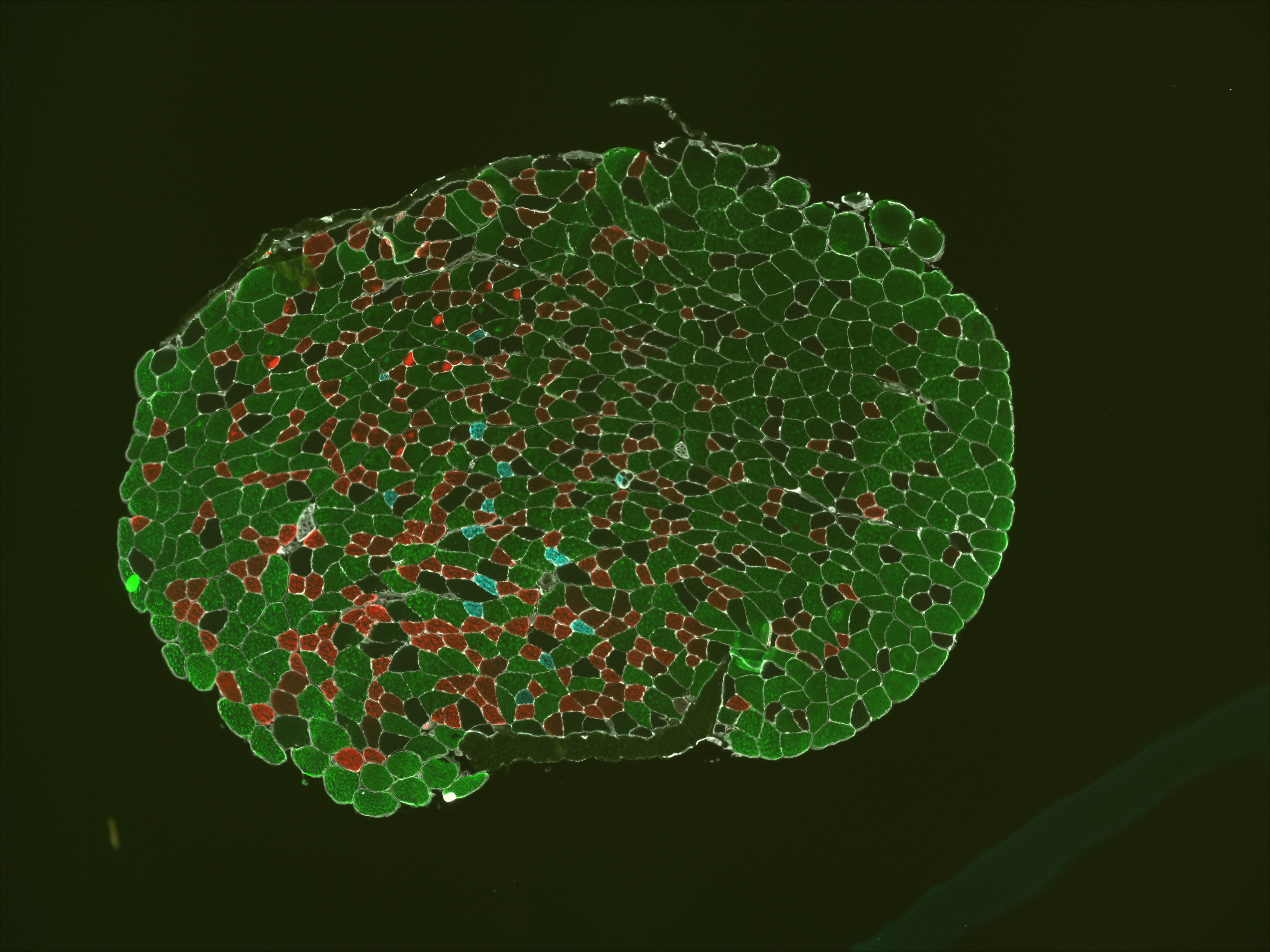

Supplement: Supplementary file 1 [file cells-10-02459-s001.zip › Supplemental Methods/Fiber Cross-Sectional Area Pipeline/Sample Images for Fiber CSA Measurements/Whole Cross-Section Images Merged.tif]

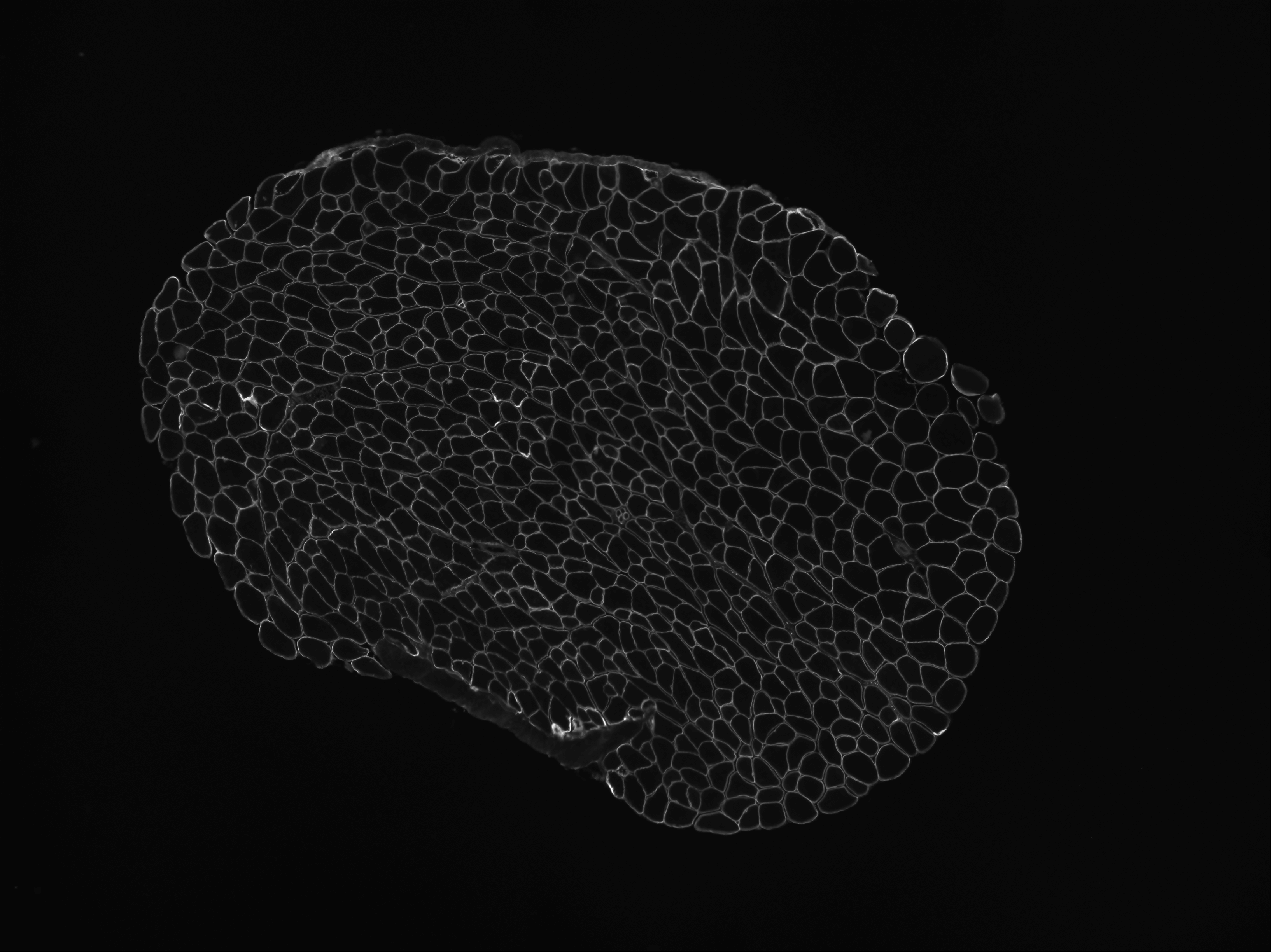

Supplement: Supplementary file 1 [file cells-10-02459-s001.zip › Supplemental Methods/Myonuclei Pipeline/Sample Images for Myonuclei Measurements/Ch0.tif]

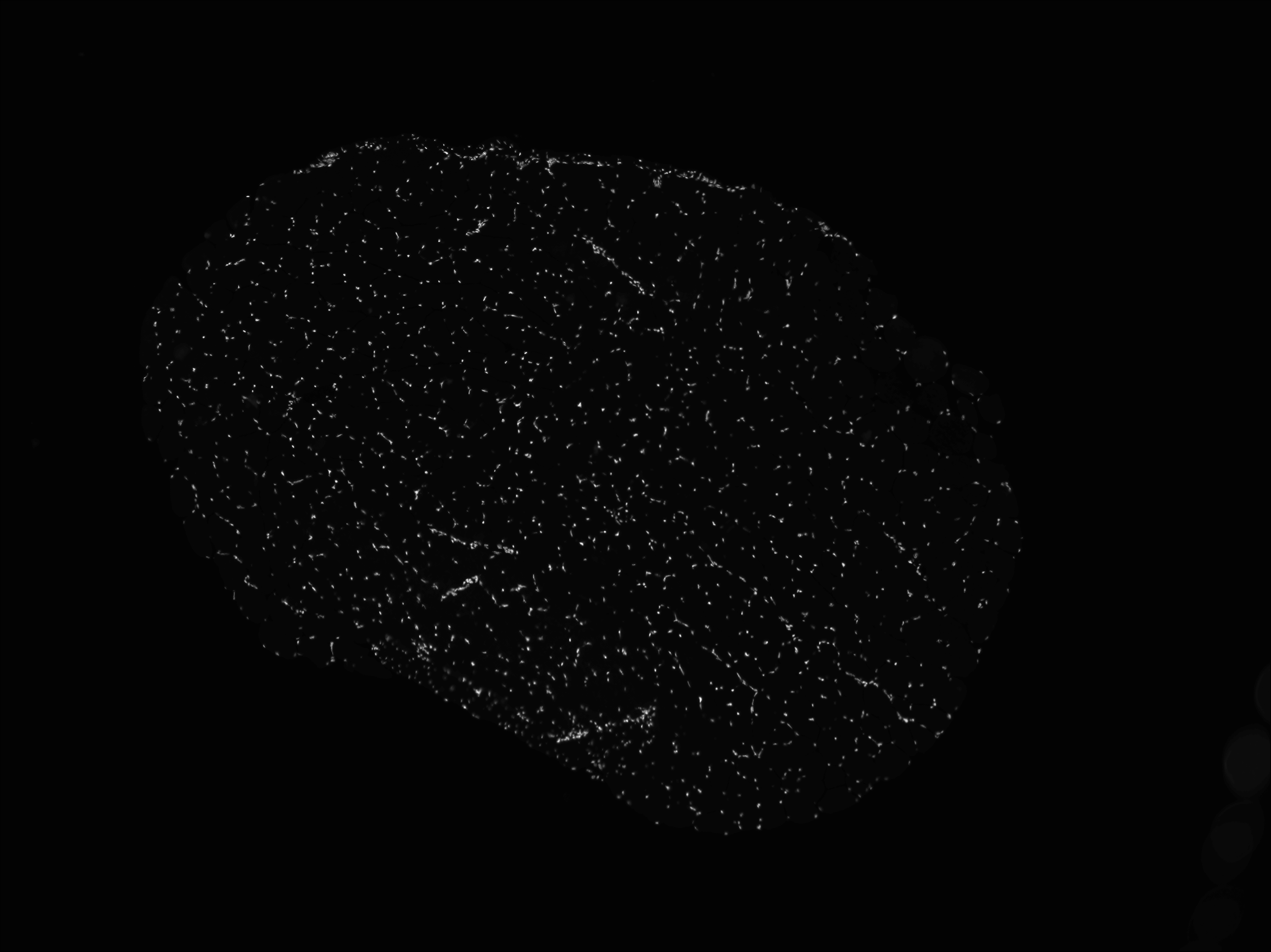

Supplement: Supplementary file 1 [file cells-10-02459-s001.zip › Supplemental Methods/Myonuclei Pipeline/Sample Images for Myonuclei Measurements/Ch1.tif]

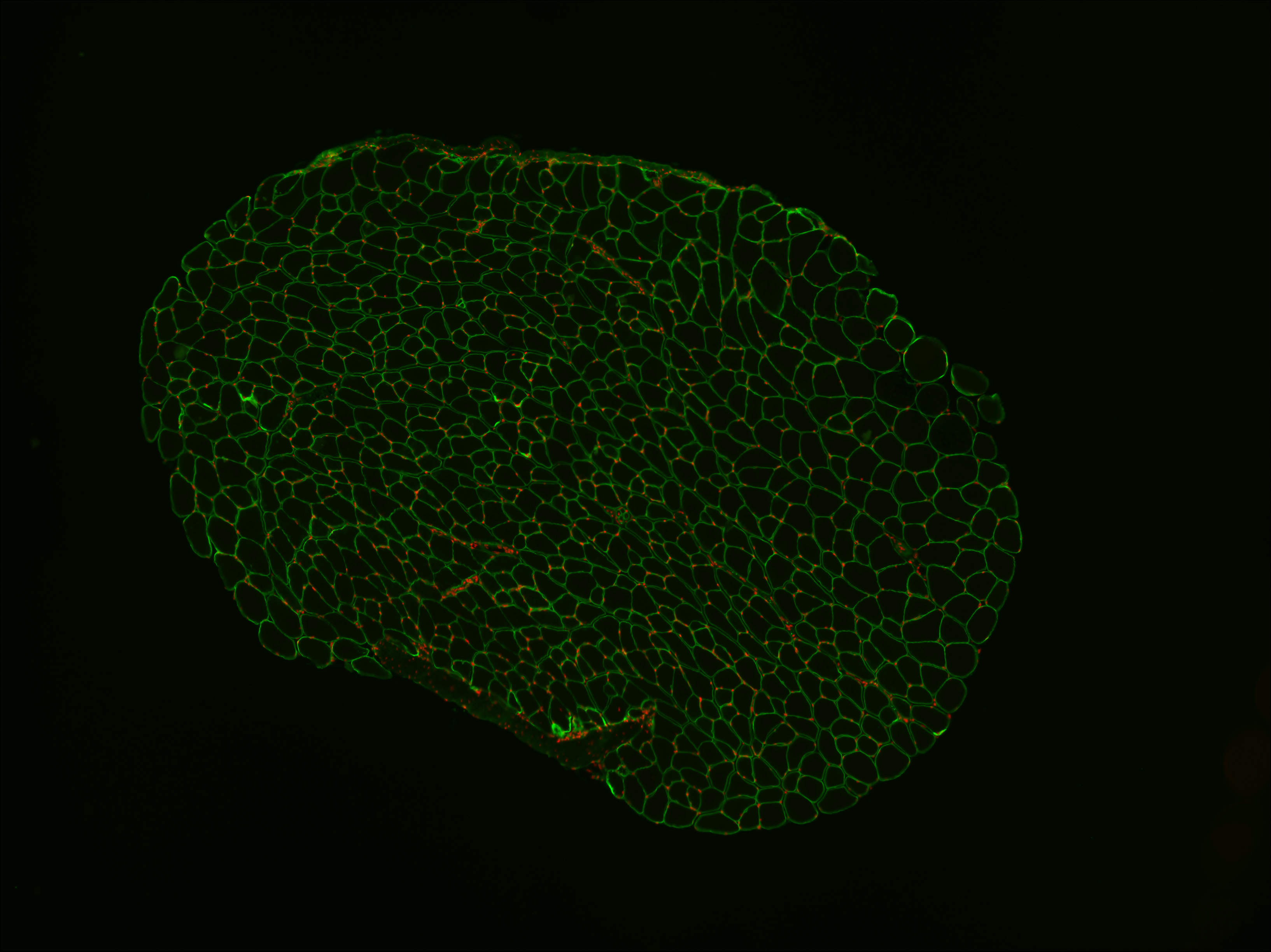

Supplement: Supplementary file 1 [file cells-10-02459-s001.zip › Supplemental Methods/Myonuclei Pipeline/Sample Images for Myonuclei Measurements/Whole Cross-Section Images Merged.tif]
